# Antigenic response of bovine β-lactoglobulin influenced by ultra-high pressure treatment in combination with high temperature

**DOI:** 10.1186/2045-7022-5-S3-P49

**Published:** 2015-03-30

**Authors:** Janire Orcajo, Iñigo Martinez de Marañon, Maria Lavilla

**Affiliations:** 1 Fundacion Azti-Tecnalia Fundazioa, Derio, Spain

## 

β-lactoglobulin (β-lg), the main whey protein fraction in bovine milk is a major allergen causing cow's milk allergy. Immunological investigations of β-lg have reported several IgE-binding regions of the protein that were linked with allergic reaction. Most IgE epitopes are specific for surface features of the protein. However, no clear association of structural feature or function with allergenicity has been established.

Heat treatment has been used as a means to influence the functional properties (gelation, emulsification…) of β-lg and it has been proposed as a means to reduce milk protein allergenicity. Although pressure -and temperature- induced denaturation of β-lg has also been used for modification of the functional properties of the protein, there is a lack of information about the antigenicity as a marker for the allergenic status of β-lg. Then, the objective of this study is to determine the modification in IgE binding to β-lg when applying High Pressure (HP), High Temperature (HT) and the combination of both technologies, HPHT.

Samples of β-lg from bovine milk in several media at different pH were pressurized at 600 MPa in a range time of 0 to 6 minutes at 25, 75 and 95^o^ C. Concurrently, same samples were treated only by heat treatment at 75 and 95^o^ C for same times. Then, an *in vitro* test by indirect competitive enzyme-linked immunosorbent assay (ELISA) with recombinant monoclonal IgE was performed in order to determine the alterations produced in the specific antigen-antibody immunoreactivity.

The experiments showed little changes in the allergenicity of the β-lg when heat treatments or HP at room temperature were applied. Nevertheless, results of combined HPHT treatments at 600 MPa and temperatures thereupon 75^o^ C clearly affect the antigenity of β-lg. In addition, beyond pressure and holding time temperature is an important parameter to modulate the antigenity of this allergen.

HPHT treatment should be considered as an alternative processing technology for reduction of allergenicity especially in the field of milk and milk products with high allergy risks that can even improve the nutritional and physic-chemical properties.

